# Supplementary material for: The First Complete Chloroplast Genome of Campanula carpatica: Genome Characterization and Phylogenetic Diversity
Source: Genes (Basel). 2023 Aug 7;14(8):1597. doi: 10.3390/genes14081597 (PMC10454809; doi:10.3390/genes14081597)
Supplement: Supplementary file 1 [file genes-14-01597-s001.zip › genes-2518457-supplementary.pdf]

Supplementary Figure S1. Schematic of the ten *cis*-splicing genes in the *C. carpatica* chloroplast genome. The genes are arranged from top to bottom on the *C. carpatica*. genome. The gene structures showed that exon regions are displayed in black color box while introns are indicated in white. The arrow indicates the sense direction of the gene.

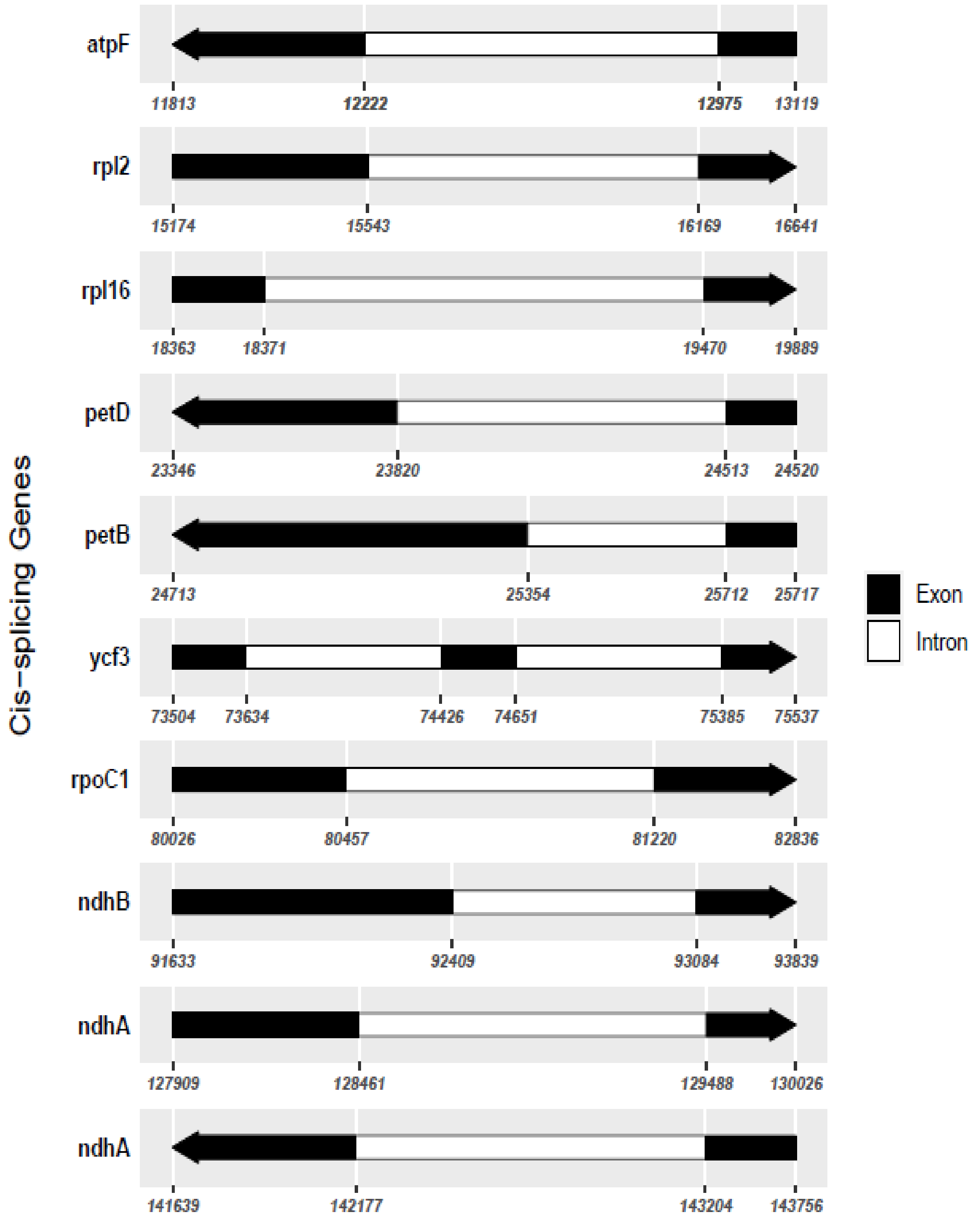

Supplementary Table S1. Simple Sequence Repeats (SSRs) in *C. carpatica.*

Number	Type	Structure	Size(bp)	Start	End
1	p1	(A)10	10	5494	5503
2	p1	(A)11	11	39123	39133
3	p1	(A)12	12	58790	58801
4	p1	(T)12	12	60184	60195
5	p1	(A)10	10	64218	64227
6	p1	(T)11	11	75583	75593
7	p1	(A)11	11	85135	85145
8	p1	(A)12	12	129074	129085
9	p1	(A)13	13	132025	132037
10	p1	(T)12	12	142580	142591
11	c*	(T)8_(A)10_(A)8	49	352	400
12	c	(T)9(G)10_(AT)5(A)8	65	26972	27036
13	c	(A)8_(GAA)4_(GAA)4_(A)12	126	112873	112998
14	c	(T)12_(TTC)4_(CTT)4_(T)8	126	158667	158792

* compound SSRs

Supplementary Table S2. Tandem repeat sequences in *C. carpatica.*

Start	End	P[1] Size	Copy Num.	C[2] Size	Match[3]	Indels	Score	Percent composition				Entropy (0-2)
								A	C	G	T	
6131	6180	25	2	25	100	0	100	52	12	24	12	1.72
6718	6981	118	2.3	120	84	5	346	26	25	23	24	2
18678	18778	44	2.3	43	88	5	141	42	19	16	20	1.89
19288	19319	16	2	16	100	0	64	37	12	0	50	1.41
26450	26599	70	2.1	70	90	3	230	26	21	12	40	1.89
29558	29630	24	3.3	23	67	21	66	43	6	19	30	1.76
29554	29766	42	5	42	91	3	329	41	7	20	30	1.81
29554	29766	85	2.5	84	88	4	329	41	7	20	30	1.81
32502	32567	24	3	21	81	12	87	30	12	25	31	1.92
33573	33598	11	2.4	11	100	0	52	42	11	11	34	1.77
34996	35051	28	2	28	96	0	103	26	14	10	48	1.76
36740	36844	42	2.5	42	95	0	183	41	8	20	28	1.82
38712	38770	20	3	20	97	0	109	61	1	13	23	1.42
44712	44793	23	3.7	23	85	6	114	47	6	17	29	1.71
56758	56857	48	2.1	48	96	0	182	39	13	13	35	1.83
56863	56922	19	3.3	19	68	13	63	46	10	10	33	1.71
57603	57666	28	2.3	28	97	2	121	35	12	25	26	1.91
58972	59003	15	2.1	15	100	0	64	21	0	31	46	1.52
67315	67344	15	2	15	100	0	60	33	13	6	46	1.69
72857	72898	17	2.5	17	96	0	75	64	4	11	19	1.44
76474	76509	17	2.1	17	89	0	54	27	19	22	30	1.98
90714	90755	21	2	21	95	0	75	47	11	11	28	1.76
95273	95366	15	6.3	15	93	0	143	42	8	45	3	1.5
95857	96022	54	3.1	54	100	0	332	44	11	21	22	1.84
96178	96369	27	7.1	27	98	0	375	22	14	26	37	1.92
97085	97188	51	2	51	94	0	181	31	11	20	36	1.88
97171	97206	18	2	18	100	0	72	22	16	16	44	1.86
97257	97404	51	2.9	51	79	7	165	26	17	23	32	1.97
97370	97470	42	2.4	42	96	3	186	27	14	18	38	1.91
97525	97578	24	2.2	24	86	0	72	35	12	20	31	1.9
97566	97665	36	2.8	36	93	6	184	31	14	33	22	1.93
97661	97856	24	7.9	24	77	6	185	31	16	21	30	1.96
97890	97963	36	2.1	36	94	5	132	28	16	32	22	1.96
97842	98101	87	3	87	96	0	466	28	17	29	24	1.97
97977	98050	36	2.1	36	92	5	123	29	16	31	22	1.96

98016	98234	51	4.3	51	94	1	341	27	17	25	29	1.98
98754	98801	12	4	12	89	5	71	41	6	43	8	1.6
98813	98864	21	2.5	20	81	12	59	50	13	23	13	1.77
108646	108829	26	7	26	87	4	203	26	23	34	15	1.94
108646	108829	52	3.5	52	89	0	260	26	23	34	15	1.94
108661	108827	78	2.1	79	86	1	237	24	25	35	14	1.94
112869	112973	30	3.4	30	75	12	104	59	0	31	9	1.3
112888	112971	15	5.4	15	91	8	141	54	0	33	11	1.37
112998	113040	12	3.6	12	87	9	61	53	2	44	0	1.13
114673	114715	20	2.2	20	83	8	52	2	27	0	69	1
118424	118462	12	3.2	12	100	0	78	53	7	23	15	1.67
118604	118763	66	2.4	66	97	0	302	31	15	30	22	1.95
119280	119355	21	3.6	21	96	0	134	46	25	6	22	1.76
119810	119912	51	2	51	98	0	197	36	19	9	33	1.85
120235	120439	33	6.2	33	99	0	392	53	8	22	14	1.68
120427	120462	18	2	18	88	0	54	61	8	19	11	1.54
120805	120906	9	11.7	9	80	10	106	66	0	33	0	0.92
120812	120909	27	3.8	26	94	4	171	63	0	36	0	0.95
121564	121680	21	5.6	21	95	0	189	41	11	20	26	1.85
122321	122356	18	2	18	100	0	72	55	11	16	16	1.68
122433	122534	27	3.8	27	81	0	123	46	13	13	26	1.81
122433	122475	15	3.1	15	74	19	56	44	13	11	30	1.8
122555	122604	24	2.1	24	100	0	100	62	12	16	10	1.55
123161	123207	15	3.1	15	100	0	94	80	0	19	0	0.7
123159	123235	15	4.4	19	84	15	80	77	3	15	2	1.02
124124	124382	26	9.7	26	90	5	410	25	24	34	15	1.94
124124	124382	81	3.2	81	93	2	443	25	24	34	15	1.94
124124	124382	107	2.4	105	93	4	430	25	24	34	15	1.94
126508	126620	57	2	57	94	0	199	51	9	17	21	1.74
130597	130650	18	3	18	91	0	90	38	5	16	38	1.72
132125	132169	20	2.2	20	100	0	90	51	4	20	24	1.66
133804	133844	20	2	20	90	9	66	53	24	0	21	1.46
137300	137343	19	2.3	19	100	0	88	50	4	15	29	1.64
141015	141068	18	3	18	91	0	90	38	16	5	38	1.72
145045	145157	57	2	57	94	0	199	21	17	9	51	1.74
145614	145658	18	2.5	19	78	10	58	11	20	4	64	1.42
147283	147541	26	9.7	26	90	5	410	15	34	24	25	1.94
147283	147541	81	3.2	81	93	2	443	15	34	24	25	1.94

147283	147541	107	2.4	108	93	4	436	15	34	24	25	1.94
148430	148506	15	4.4	19	81	18	80	2	15	3	77	1.02
148458	148504	15	3.1	15	100	0	94	0	19	0	80	0.7
149061	149110	24	2.1	24	100	0	100	10	16	12	62	1.55
149131	149232	27	3.8	27	81	0	123	26	13	13	46	1.81
149190	149232	15	3.1	15	74	19	56	30	11	13	44	1.8
149309	149344	18	2	18	100	0	72	16	16	11	55	1.68
149985	150101	21	5.6	21	95	0	189	26	20	11	41	1.85
150759	150860	9	11.7	9	80	10	106	0	33	0	66	0.92
150756	150853	27	3.8	26	94	4	171	0	36	0	63	0.95
151203	151238	18	2	18	88	0	54	11	19	8	61	1.54
151226	151430	33	6.2	33	99	0	392	14	22	8	53	1.68
151753	151855	51	2	51	98	0	197	33	9	19	36	1.85
152310	152385	21	3.6	21	96	0	134	22	6	25	46	1.76
152902	153061	66	2.4	66	97	0	302	22	30	15	31	1.95
153203	153241	12	3.2	12	100	0	78	15	23	7	53	1.67
155230	155262	16	2.1	16	88	5	50	18	39	15	27	1.9
158625	158667	12	3.6	12	87	9	61	0	44	2	53	1.13
158696	158782	33	2.8	31	86	8	115	11	32	0	56	1.35
158694	158777	15	5.4	15	95	4	141	11	33	0	54	1.37
162836	163019	26	7	26	89	0	242	15	34	23	26	1.94

* [1)] P size: Period Size(bp), [2)] C size: Consensus Size(bp), [3)] Match: Percent Matches

Supplementary Table S3. Dispersed repeat sequences in *C. carpatica.*

First length	First start	Repeat type*	Second length	Second start	Interval Distance	E-value
781	30399	D	781	37477	-3	0.00E+00
772	30408	D	772	37486	-2	0.00E+00
513	28490	D	513	111373	-3	0.00E+00
513	28490	P	513	159778	-3	0.00E+00
496	76035	D	496	115210	-2	2.13E-283
496	76035	P	496	155958	-2	2.13E-283
500	30346	D	500	37424	-3	4.21E-283
482	30748	D	482	125640	0	5.17E-281
482	30748	P	482	145542	0	5.17E-281
482	30375	D	482	37453	-3	2.59E-272
469	37826	D	469	125640	-3	1.60E-264
469	37826	P	469	145555	-3	1.60E-264
369	29661	D	369	36739	0	5.58E-213
377	28363	D	377	111246	-3	2.04E-209
377	28363	P	377	160041	-3	2.04E-209
370	30847	D	370	37925	-2	8.57E-208
370	37925	D	370	125739	-2	8.57E-208
370	37925	P	370	145555	-2	8.57E-208
359	30858	D	359	37936	-1	6.30E-204
359	37936	D	359	125750	-1	6.30E-204
359	37936	P	359	145555	-1	6.30E-204
330	28673	D	330	111556	-2	8.24E-184
330	28673	P	330	159778	-2	8.24E-184
276	31215	D	276	116953	-2	1.87E-151
276	31215	P	276	154435	-2	1.87E-151
262	28741	D	262	111624	-1	1.15E-145
262	28741	P	262	159778	-1	1.15E-145
219	31272	D	219	117010	-1	7.47E-120
219	31272	P	219	154435	-1	7.47E-120
227	36322	D	227	58221	-3	9.01E-120
210	31281	D	210	117019	0	2.98E-117
210	31281	P	210	154435	0	2.98E-117
209	25415	P	209	112254	-2	2.33E-111
209	25415	D	209	159201	-2	2.33E-111
190	36370	D	190	58269	-3	9.95E-98

180	120226	D	180	120259	-3	8.86E-92
180	120226	P	180	151225	-3	8.86E-92
180	120259	P	180	151258	-3	8.86E-92
180	151225	D	180	151258	-3	8.86E-92
165	96177	D	165	96204	-2	4.49E-85
161	64320	D	161	124448	-1	4.56E-85
161	64320	P	161	147055	-1	4.56E-85
151	97841	D	151	97928	-3	1.50E-74
144	151265	D	144	151298	-3	2.13E-70
143	120234	D	143	120300	-3	8.36E-70
143	120234	P	143	151221	-3	8.36E-70
143	120300	P	143	151287	-3	8.36E-70
143	151221	D	143	151287	-3	8.36E-70
130	30975	D	130	36609	0	4.35E-69

*Repeat type (D: direct matches; P: palindromic matches)
